# Field-road classification for agricultural vehicles in China based on pre-trained visual model

**DOI:** 10.7717/peerj-cs.2359

**Published:** 2024-10-16

**Authors:** Xiaoqiang Zhang, Ying Chen

**Affiliations:** 1 College of Information and Electrical Engineering, China Agricultural University, Beijing, China; 2 Key Laboratory of Agricultural Machinery Monitoring and Big Data Applications, Ministry of Agriculture and Rural Affairs, Beijing, China

**Keywords:** Field-road classification, Pretraining-finetuning paradigm, Image recognition, Multi-view sequential learning

## Abstract

Field-road classification that automatically identifies the activity (either in-field or on-road) of each point in Global Navigation Satellite System (GNSS) trajectories is a critical process in the behavior analysis of agricultural vehicles. To capture movement patterns specific to agricultural operations, we propose a multi-view field-road classification method, which extracts a physical and a visual feature vector to represent a trajectory point. We propose a task-specific approach using a pre-trained visual model to effectively extract visual features. Firstly, an image is generated based on a point plus its neighboring points to provide the contextual information of the point. Then, an image recognition model, a fine-tuned ResNet model is developed using the pretraining-finetuning paradigm. In such a paradigm, a pre-training process is used to train an image recognition model (ResNet) with natural image datasets (*e.g.*, ImageNet), and a fine-tuning process is applied to update the parameters of the pre-trained model using the trajectory point images, enabling the model to have both general knowledge and task-specific knowledge. Finally, a visual feature is extracted for a point by the fine-tuned model, thereby overcoming the limitations caused by the small-scale generated images. To validate the effectiveness of our multi-view field-road classification, we conducted experiments on four trajectory datasets (Wheat 2021, Paddy, Wheat 2023, and Wheat 2024). The results demonstrated that the proposed method achieves competitive accuracy performance, *i.e*., 92.56%, 87.91%, 90.31%, and 94.23% on four trajectory datasets, respectively. Extensive experiments demonstrate that our approach can consistently perform better than the existing state-of-the-art method on the four trajectory datasets by 2.99%, 4.42%, 2.88%, and 2.77% in the F1-score, respectively. In addition, we conduct an in-depth analysis to verify the necessity and effectiveness of our method.

## Introduction

With the popularization and development of vehicle Global Navigation Satellite System (GNSS) technology, a large number of GNSS trajectories have been generated during various agricultural vehicle driving. The accumulation of GNSS trajectory data gives rise to various activity classification tasks that classify the activity of trajectory points (*e.g.*, driving on the road, working in the field). Activity analysis is very crucial for agricultural vehicles, as it helps in understanding various agricultural activities (Kortenbruck, Griepentrog & Paraforos, 2017; Hanke, Trösken & Frerichs, 2018), improving optimized operation plans for the efficient use of agricultural vehicles (Utamima, Reiners & Ansaripoor, 2019; Jeon et al., 2021), and providing a real-time activity monitor for making informed decisions by authorities (Shi, Paudel & Chen, 2021).

**Figure 1:**
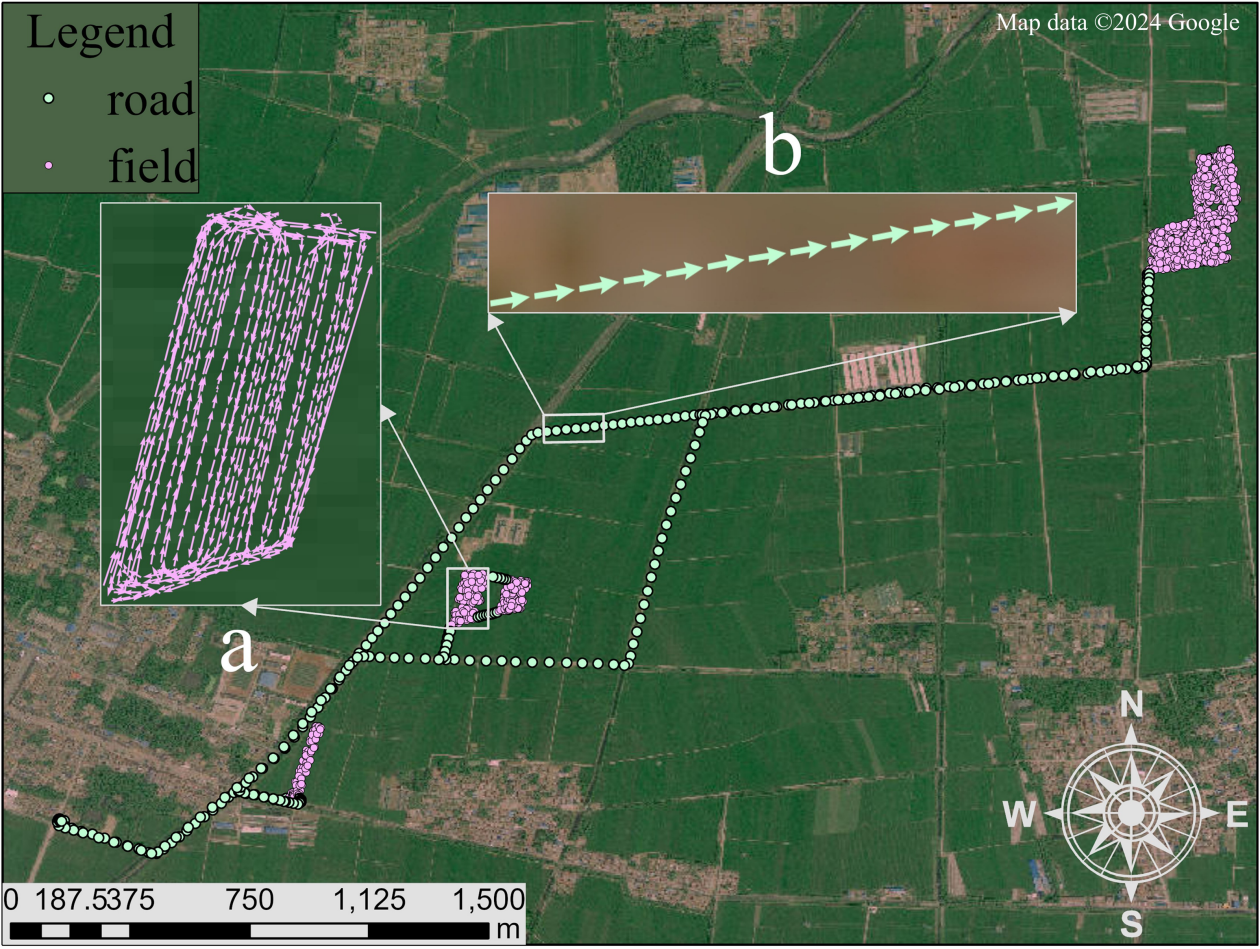
An example trajectory of an agricultural vehicle, illustrating the distinction between field and road segments. (A) Field segment: This sub-trajectory depicted with pink points follows a pattern characteristic of agricultural operations, comprising alternating straight trips and turning curves. The straight trips indicate operational passes within the field, while the curves represent turns at the end of each pass. (B) Road segment: This sub-trajectory depicted with green points represents the vehicle’s movement on the road, characterized by a more linear and continuous path without the alternating pattern seen in field operations. Satellite imagery© 2024 Google Earth Engine; Map data© 2024 Google.

In this paper, we work on the first step of activity analysis for agricultural vehicles, field-road classification that distinguishes in-field activities (namely “field” segments) and out-of-field activities (namely “road” segments) in a GNSS trajectory recorded during an agricultural vehicle driving, as shown in Fig. 1. Here, a segment refers to continuous trajectory points with the same activity, and either field segments or road segments can be used to make a further activity analysis, *e.g.*, harvesting and turning in a field trajectory segment (Bochtis et al., 2010; Duttmann, Brunotte & Bach, 2013). Due to the unavailability of external resources (*e.g.*, a field boundary map) (Yang et al., 2013; Zhang, Yang & Thomas, 2017), field-road classification often relies exclusively on GNSS trajectories, which requires the detection of specific movement patterns. As observed in previous studies (Zhai et al., 2024; Poteko, Eder & Noack, 2021; Chen et al., 2021), there are two motion characteristics: (1) physical motion, *i.e.,* motion parameters (*e.g.*, speed, acceleration) are different in fields and on road; (2) travel shape in a field, *i.e.,* the sub-trajectory in a field is often composed of strips and turning curves alternately, as shown in Fig. 1A. This paper targets the capture of motion and shape characteristics for the improvement of field-road classification performances.

Field-road classification methods have made some progress, which can be divided into two categories: clustering-based and classification-based. The former clusters trajectory points based on their density to form clusters of fields and roads (Chen et al., 2021; Zhang et al., 2022), and the latter uses the feature representation of each trajectory point to perform a binary classification (either “field” or “road”) for the point. Due to its superior performance, the classification-based method has often been a choice in previous studies, and its key research issue is the feature representation of each trajectory point. To detect the physical motion characteristic, Poteko, Eder & Noack (2021) and Zhai et al. (2024) have used the statistics of motion parameters in the region where a trajectory point is located as its feature representation. To capture the shape characteristics in a field, several deep learning methods have been developed. For example, Chen et al. (2022) proposed a graph convolutional neural network to extract the spatio-temporal information in the neighborhood of a point. Chen et al. (2023) proposed an image segmentation method (including image generation and visual feature extraction) to capture pixel-level features in an image to support trajectory point classification. The field-road classification method using visual features has been proven to perform best (Chen et al., 2023), but the shape characteristic in a field still have not been effectively captured through the visual features due to the following two problems: (1) Detailed information around each point (*i.e.,* spatial location information of each trajectory point and its adjacent points) has not been well presented in the image generated in Chen et al. (2023). As only one image is automatically generated for each trajectory, the image is too coarse-grained to encode so much detailed information. (2) The visual features of a point have not been effectively extracted using the limited image segmentation model developed in Chen et al. (2023). The limited model capacity is because the used training data (*i.e.,* manually-labeled field-road classification data) is small-scale.

To tackle the first problem, instead of the trajectory-based image generation used in Chen et al. (2023), we propose a point-based image generation method which generates an image for each trajectory point. The method encodes the context of a point as a colorful image, where the context refers to the information provided by the point and its neighboring points (*e.g.*, the shape formed by the points, and the speed of the points). By generating an image for each trajectory point, the detailed information around each point can be well presented.

To solve the second problem, we adopt the pretraining-finetuning paradigm (Chu et al., 2016; Tajbakhsh et al., 2016) to develop an image recognition model for visual feature extraction. In such a paradigm, an existing powerful pre-trained image recognition model is fine-tuned for our field-road classification task, and then visual features are extracted from our point-based images using the fine-tuned model. Such a paradigm provides a way to utilize general knowledge that has been learned by the pre-trained model to help our visual feature extraction (a task-specific knowledge-learning problem). Through using the rich knowledge in the pre-trained model, the impact of data limitations can be mitigated.

Specifically, the multi-view input feature extraction is used to represent a trajectory point by two feature vectors: physical feature vector and visual feature vector. The physical feature vector is extracted from a physical view, which provides the physical motion characteristics (*e.g.*, speed, direction, and acceleration) of the point. The visual feature vector is extracted from a visual view, which gives the shape characteristic of an image generated by the point and its neighboring points. Notice that the two feature vectors are extracted independently of each other. Then, Bi-directional Long Short-Term Memory (BiLSTM) (Graves & Schmidhuber, 2005), a deep neural network, is used to extract a multi-view fusion feature vector for each point based on its two feature vectors. Finally, a point is classified with its multi-view fusion feature vector. Experiments on four field-road classification benchmark datasets demonstrate the effectiveness of our multi-view field-road classification approach.

Our main contributions to the field-road classification task can be summarized as follows:

• We proposed a multi-view field-road classification method that can fuse physical and visual feature vectors to represent the detailed information of trajectory points.
• We proposed a point-based image generation method to present detailed information around each trajectory point, and adopted the pretraining-finetuning paradigm to utilize general and task-specific visual knowledge for visual feature extraction.
• We constructed two new trajectory datasets, collected in the last two years (*i.e.,* 2023 and 2024), which can enrich the field-road trajectory dataset.
• Experimental evaluations on four datasets with varying-quality trajectories demonstrate the effectiveness and robustness of our proposed method.

## Methods

### Datasets

The proposed methodology was trained and tested on the four trajectory datasets collected by wheat harvesters and paddy harvesters in China. The four trajectory datasets include a wheat harvesting trajectory dataset collected in 2021 (Wheat 2021), a paddy harvesting trajectory dataset collected in 2021 (Paddy), a wheat harvesting trajectory dataset collected in 2023 (Wheat 2023), and a wheat harvesting trajectory dataset collected in 2024 (Wheat 2024). Furthermore, Wheat 2021 and Paddy are collected by Chen et al. (2022).

Each harvester was installed with a GNSS positioning terminal, and the positioning accuracy was 5 m. Once a harvester got started in a day, a daily trajectory began to be recorded. Each point of a trajectory contains five parameters: timestamp (YYYY:MM:DD-hh: mm:ss), longitude (°), latitude (°), speed (m/s), and direction (°). As shown in Table 1, the four datasets are different in data statistics, such as recording updated frequency (5s *vs.* 30s for the wheat and paddy harvesting trajectories). Moreover, the trajectories of the datasets are spatially distributed across fourteen provinces of China, from north regions to south regions.

**Table 1:** Statistics of the four datasets.

Dataset	Wheat 2021	Paddy	Wheat 2023	Wheat 2024
# trajectory	150	100	30	30
# trajectory points	995,946	126,602	161,428	195,738
Max length	17,233	4,410	23,404	39,027
Min length	539	214	1,887	1,983
Average length	6,639	1,266	5,381	6,525
Frequency	5 s	30 s	5 s	5 s
Acquisition period	2021.06	2021.10	2023.06	2024.06

**Notes.**

*length* indicates the number of points in a trajectory.

Furthermore, for each trajectory, existing data preprocessing (Chen et al., 2021) was applied, which includes smoothing noise points and removing duplicated points. The processed data were manually annotated, and each point of a trajectory was marked as either “field” or “road”. Moreover, each dataset is randomly divided into the training set and test set according to the ratio of 9:1. The datasets are available at: https://zenodo.org/records/10561232.

### Overview

**Figure 2:**
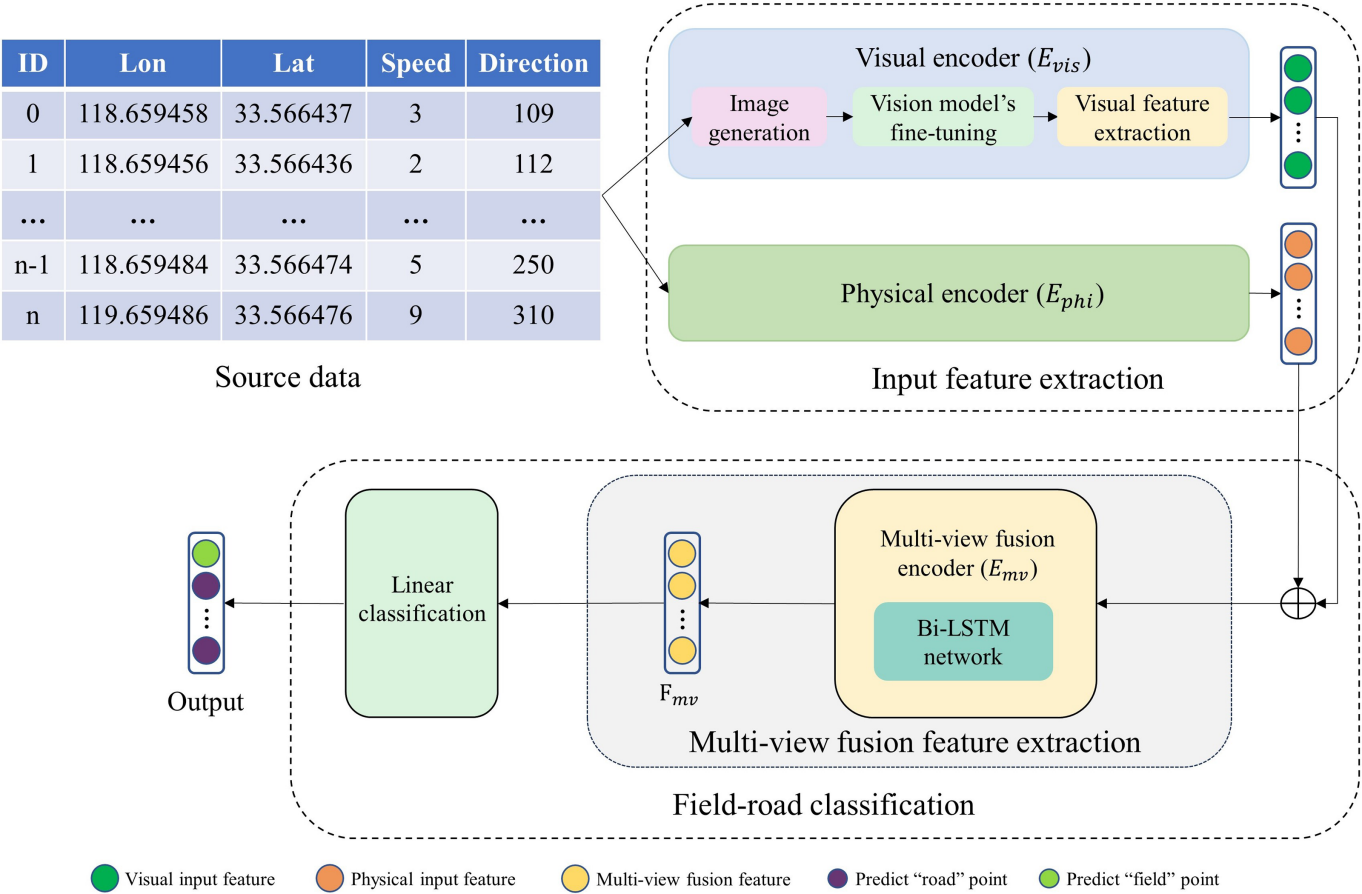
The proposed multi-view sequence learning field-road classification method. ‘⊕’ represents concatenation.

As illustrated in Fig. 2, our multi-view field-road classification framework comprises three parts: input feature extraction, multi-view fusion feature extraction, and linear classification. The uni-view feature extraction (Visual encoder and Physical encoder) provides input features to field-road classification consisting of the multi-view fusion feature extraction and the linear classification.

(1) **Input feature extraction**: For each point in a trajectory, two uni-view encoders (physical encoder *E*_*phi*_ and visual encoder *E*_*vis*_) are applied to extract two input feature vectors (a physical feature vector and a visual feature vector) from the physical view and visual view, respectively. The physical feature provides the physical motion characteristics, and the visual feature gives the shape characteristic. Moreover, in encoder *E*_*vis*_, an image is generated for each point, and then a visual feature is extracted from the image using an image recognition model which has been developed through fine-tuning the pre-trained ResNet model.

(2) **Multi-view fusion feature extraction**: a multi-view fusion encoder *E*_*mv*_ is used to extract a multi-view fusion feature vector *F*_*mv*_ for each point based on its two input feature vectors.

(3) **Linear classification**: a point is classified using a linear layer based on its *F*_*mv*_.

### Physical encoder

We follow the feature extraction used by Chen et al. (2022) to extract the physical motion characteristics of a point. Specifically, the physical encoder *E*_*phi*_ represents a point as a vector with seven features (speed, longitude_diff, latitude_diff, direction_diff, acceleration, jerk, bearing rate). The speed is the recorded speed on the point. Longitude_diff, latitude_diff, and direction_diff are the differences in the longitude, latitude, and direction between the current point and its previous point, respectively. Acceleration, jerk, and bearing rate are given in Dabiri & Heaslip (2018).

### Visual encoder

As illustrated in Fig. 2, there are three components in the visual encoder *E*_*vis*_: image generation, vision model’s fine-tuning, and visual feature extraction. The image generation module creates an image for each trajectory point, containing the contextual information specific to that point (*e.g.*, several parallel strips, the speed of the point and its neighboring points). Vision model’s fine-tuning provides an image recognition model through fine-tuning the existing pre-trained ResNet model (He et al., 2016). Based on the fine-tuned ResNet model, the visual feature extraction module extracts a visual feature vector for each point in a trajectory.

**Figure 3:**
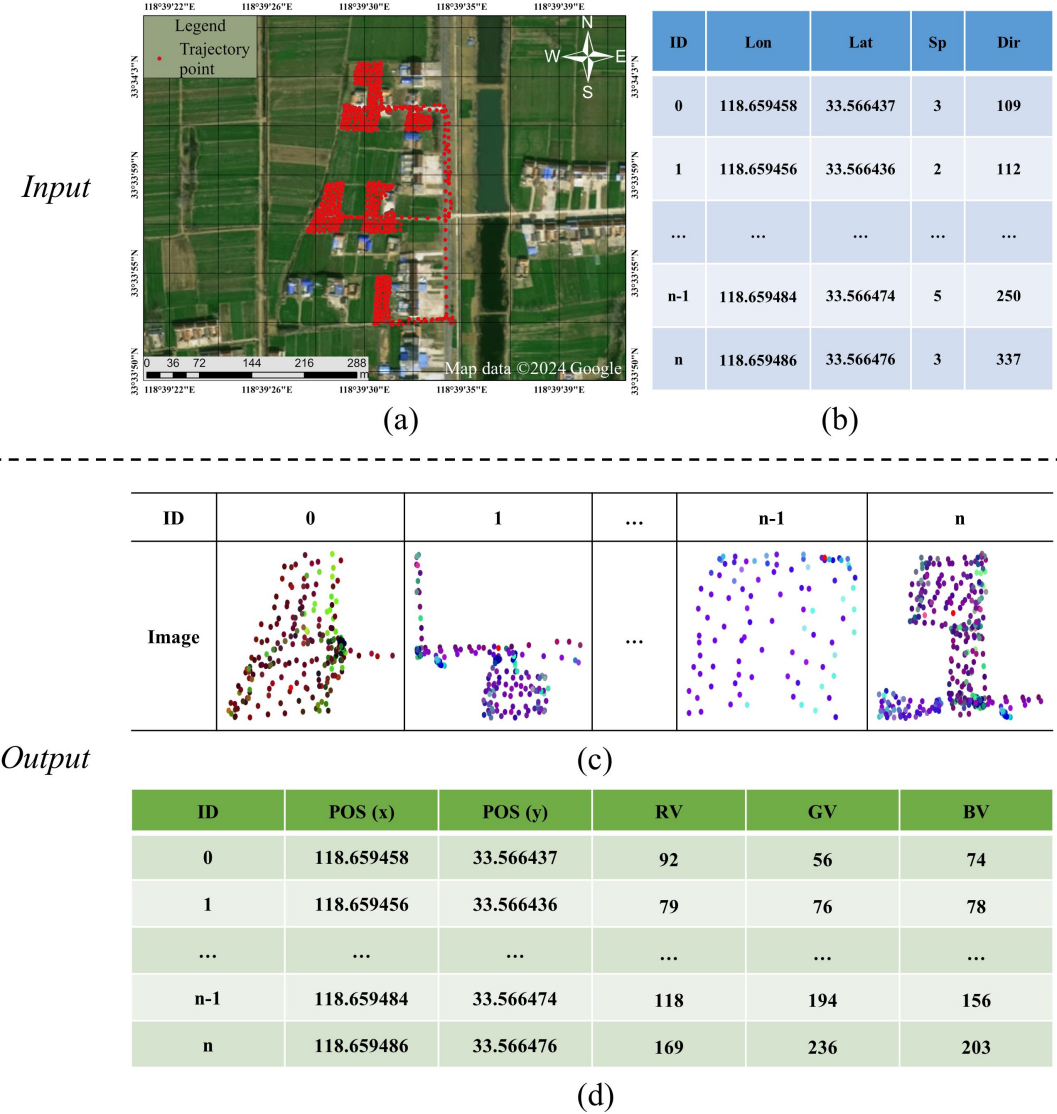
An example of image generation: input is (B) and output is (D). (A) A trajectory contains n points; (B) the trajectory data: longitude (lon), latitude (lat), speed (sp), and direction (dir); (C) images: there are n images for n points, respectively; (D) the data representation of an image: pixel position (POS), red value (RV), green value (GV) and blue value (BV). Satellite imagery© 2024 Google Earth Engine; Map data© 2024 Google.

Image generation: In order to capture the shape formed by a point and its neighboring points, we generate a 2D image for the point, and such an image contains points located in a rectangle region of the point. For example, n images (Fig. 3C) are generated for n points in a trajectory (Fig. 3A) one by one, where each point is represented by a row in Fig. 3B, and each image is represented by the format of Fig. 3D. Since both all images input to the ResNet model must be the same-sized pixels in either dimension and the distance information in a trajectory needs to be kept. We follow the work of Endo et al. (2016) to clip a fix-sized rectangle region centered on the point, where a rectangle region is defined by ranges of latitude and longitude (the side length of the rectangle is 0.0002° in this study). Then, following the image generation method proposed by Chen et al. (2023), we generate a colorful image for each rectangle region, where the three color channels (Red-Green-Blue; RGB) are used to encode three physical motion characteristics (speed, direction, speed+direction), respectively. Specifically, the value in the R channel is the ratio of the speed at the current point to the maximum speed in the entire trajectory, the value in the G channel is the ratio of the direction at the current point and 360° (the maximum direction value), the value in the B channel is the average of the values in the R and G channel, the calculated formulas of R, G, and B as Eqs. (1)–(3). Notice that unlike an image per point in our method, Chen et al. (2023) generated an image for the whole trajectory, and the detailed information on each point is missing in such an image.

(1) \begin{eqnarray*}R= \frac{speed(i)}{max(speed)} \end{eqnarray*}

(2) \begin{eqnarray*}G= \frac{direction(i)}{360} \end{eqnarray*}

(3) \begin{eqnarray*}B= \frac{R+G}{2} \end{eqnarray*}

where *speed*(*i*) is the speed of *i*-*th* trajectory point, *max*(*speed*) is the maximum speed in the entire trajectory, *direction*(*i*) is the direction of *i*-*th* trajectory point, and 360 represents all directional degrees in a two-dimensional coordinate system.

**Figure 4:**
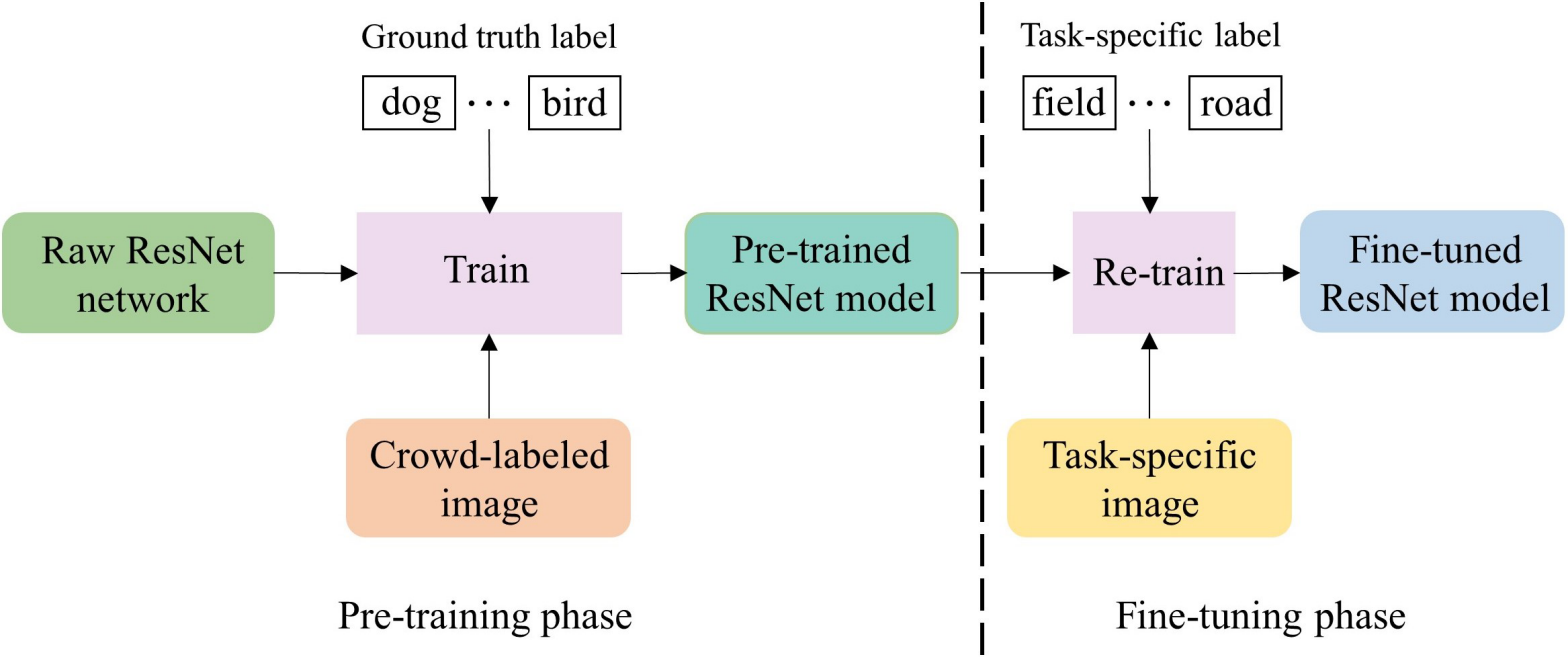
The process of fine-tuning ResNet model.

Vision model’s fine-tuning: the significant progress of the pretraining-finetuning paradigm has achieved great success in the vision understanding domain (Chu et al., 2016; Tajbakhsh et al., 2016), which consists of two phrases, as shown in Fig. 4. In the pre-training phase, a visual model is trained based on a deep neural network architecture and large-scale high-quality natural images. Moreover, such a visual model usually can effectively learn the general knowledge in natural images. In the fine-tuning phase, the pre-trained ResNet model is loaded, and the model parameters are re-trained with task-specific images so that the model can learn the task-specific knowledge in the images. To utilize both the rich general knowledge and the task-specific knowledge, we adopt this paradigm for our image recognition model development.

As illustrated in Fig. 4, we choose the pre-trained ResNet, an image recognition model which has been trained using the raw ResNet network and large-scale crowd-labeled images (Deng et al., 2009). Then, we fine-tune the pre-trained visual model for our specific image recognition task that assigns a label (either “field” or “road”) to each image. The data used for fine-tuning is the images generated by the image generation module for training trajectories, and the task-specific label of an image is the label of the central point in the focused training trajectory segment.

The model training involves optimizing the parameters of the ResNet neural network using the training image data. The parameter optimization in the pre-training phase is shown in Eq. (4), where the parameters of the raw ResNet network (*W*_1_) are initialized in the way used in He et al. (2016). Based on the large-scale crowd-labeled images, (*x*_1_, *y*_1_), *W*_1_ are updated to *W*_2_, producing the pre-trained ResNet model. Then, the parameter optimization in the fine-tuning phase is illustrated in Eq. (5), where the parameters *W*_2_ are updated to *W*_3_ using the task-specific images, (*x*_2_, *y*_2_), producing the fine-tuned ResNet model.

(4) \begin{eqnarray*}ResNet({W}_{1})\rightarrow _{}^{({x}_{1},{y}_{1})}ResNet({W}_{2}),{x}_{1}\in \{ natural~images\} ,{y}_{1}\in \{ natural~image~labels\} \end{eqnarray*}

(5) \begin{eqnarray*}ResNet({W}_{2})\rightarrow _{}^{({x}_{2},{y}_{2})}ResNet({W}_{3}),{x}_{2}\in \{ task\text{-}specific~images\} ,{y}_{2}\in \{ task\text{-}specific~labels\} \end{eqnarray*}

where *W*_1_ is the initialized model weights (*i.e.,* weights of the model without any training), *W*_2_ is the pre-trained model weights which were trained in natural image datasets, and the *W*_3_ is the fine-tuned model weights in the task-specific images (*i.e.,* field-road trajectory point image).

Visual feature extraction: As illustrated in Fig. 2, given the fine-tuned ResNet model, visual features are extracted for each input image. Specifically, the image is fed to the fine-tuned ResNet, and the features generated by the backbone of the fine-tuned ResNet are output as the visual features. Although there seems a big difference between our generated images (*i.e.,* task-specific images in Fig. 4) and the crowd-labeled natural images (*i.e.,* crowd-labeled images in Fig. 4) used to train the pre-trained ResNet model, we observe that the visual feature extraction is still effective. This confirms that the task-specific knowledge in our images has been learned through the vision model’s fine-tuning.

### Multi-view fusion encoder

Besides the physical and visual features, there are sequential relationships between points of a trajectory, which are also crucial for detecting the motion and shape information of a trajectory. Similar to Chen et al. (2023), BiLSTM is applied to capture such sequential relationships. BiLSTM is a kind of recurrent neural network which can effectively extract short-term and long-term information.

As illustrated in Fig. 2, for each point in a trajectory, its physical feature vector and visual feature vector are concatenated to a new feature vector, and thus the trajectory is represented by a sequence of the catenated feature vectors. Then, the sequence is fed to the BiLSTM network to obtain a new sequence of feature vectors, which provides a multi-view sequential fusion feature representation for the trajectory.

### Linear classification

Given the multi-view fusion feature vector *F*_*mv*−*i*_ for the i-th trajectory point, which is obtained from the multi-view fusion encoder, the softmax layer is used to calculate the probabilities over the categories (‘field’ and ‘road’) for the point (see Eq. (6)), and the category with a higher probability is selected as the final one (see Eq. (7)).

(6) \begin{eqnarray*}{p}_{i}=softmax({F}_{mv-i})\end{eqnarray*}

(7) \begin{eqnarray*}{y}_{i}=softmax({p}_{i})\end{eqnarray*}

where *p*_*i*_ denotes the predicted probability distribution over the two categories (*i.e.,* “field” and “road”) and the *y*_*i*_ denotes the final predicted category for the *i*-*th* point, respectively.

## Results and Discussion

### Experimental settings

**Baseline methods**: For a comprehensive evaluation of our proposed method, namely Phi+ResNet(finetune)+BiLSTM, the following four state-of-the-art field-road classification methods were chosen as baselines.

• **DBSCAN+Rules (DR)** (Chen et al., 2021): It is a method that combines a density clustering method (DBSCAN), and a set of direction-distribution-based rules designed for the field-road classification.
• **Decision Tree (DT)** (Poteko, Eder & Noack, 2021): It is a method that extracts features with 25 dimensions to represent a trajectory point and then feeds the features to a traditional supervised learning algorithm, a decision tree, to make the field-road classification.
• **Graph convolutional network (GCN)** (Chen et al., 2022): It is a graph convolutional neural network designed for field-road classification, in which a spatio-temporal feature vector is learned to represent a trajectory point. Given a trajectory, a spatio-temporal graph is constructed, and then a graph convolution process is used to extract the spatio-temporal features, which have aggregated the features of the neighbors of the point.
• **SVB** (Chen et al., 2023): The approach initially employs a computer vision model to perform semantic segmentation of images, extracting fine-grained pixel-level features of trajectory points. Then, these pixel-level features are combined with the statistical features of each trajectory point and fed into a fully connected network for field-road classification.

**Evaluation metrics**: following previous work Chen et al. (2021), Poteko, Eder & Noack (2021), we chose four metrics to evaluate the performance of the field-road classification method: the precision (Pre), recall (Rec), F1-score (F1), and accuracy (Acc). The performance on the test set is reported. The experimental results of our models are averaged over 10 runs with different random seeds to ensure the final reported results are statistically stable.

**Implementation details**: For our multi-view field-road classification method, we used the BiLSTM interface provided by PyTorch (a Python-based deep learning library) (Paszke et al., 2019), and chose the ResNet interface published by He et al. (2016) as our pre-trained ResNet model where the number of layers was set to 50. Moreover, for DBSCAN + Rules, SVB, and GCN, we directly used the implementations in the original paper, and for DT, we used the re-implementation method in Chen et al. (2022).

**Training details**: The goal of the training process is to learn model parameters in such a way that a loss function is minimized. There are two models to be learned in our method: the fine-tuned ResNet model, and the field-road classification model including the multi-view fusion encoder and the linear classification (Fig. 2). Specifically, the fine-tuned ResNet model was obtained by re-training the pre-trained ResNet model (as shown in Fig. 4). During the re-training process, each batch contained 128 images. On the other hand, the field-road classification model was trained from scratch. For the BiLSTM network, the sequence length of a segment was set to 32, and the hidden state size and layer number of BiLSTM were set to 256 and 2, respectively. During the training process, each batch contained four trajectory segments. Moreover, during either the training or re-training process, the cross-entropy loss function (Hinton, Osindero & Teh, 2006) was used to calculate the error between predicted labels and ground truth labels, the Adam optimizer (Kingma & Ba, 2014) was used to update model parameters, and the learning rate was set to 0.0001. Finally, the configurations used in our experiment are listed in Table 2.

**Table 2:** Experimental configuration.

Configuration	Parameter
CPU	Intel(R) Xeon(R) Gold 6226R CPU @ 2.90 GHz
GPU	Tesla V100-PCIE-32GB
Operating system	Ubuntu 18.04.6 LTS
Accelerated environment	CUDA 12.1
Libraries	torch:2.2.0, numpy:1.21.6

### Method comparisons

To compare the effectiveness of the baselines with our proposed method, the five field-road classification methods were evaluated on the four datasets, and the performances are shown in Table 3. From the table, we observe that our method, Phi+ResNet(finetune)+BiLSTM, has achieved the best performances on four trajectory datasets (Wheat 2021, Paddy, Wheat 2023, and Wheat 2024) with accuracy scores of 92.56%, 87.91%, 90.31%, and 94.23%, respectively. The result indicates that our multi-view fusion feature extraction can effectively capture the motion and shape characteristics for the field-road classification task. Moreover, the overall performance improvement is a result of the improvement on the two categories (“field” and “road”), as illustrated in Table 4. For example, on the Wheat 2021 trajectory dataset, compared with the best baseline method (*i.e.,* SVB), the F1-score of our method increases by 19.08% (from 58.56% to 77.64%) on the “road” category and 2.9% (from 92.60% to 95.50%) on the “field” category.

**Table 3:** The overall performances of five methods on four trajectory datasets.

Dataset	Pre	Rec	F1	Acc	Pre	Rec	F1	Acc
	Wheat 2021				Paddy			
DR	77.84	65.50	68.31	84.65	67.03	67.97	66.79	71.60
DT	68.16	52.10	49.48	81.37	65.74	65.45	65.48	71.40
GCN	80.66	72.92	75.14	86.96	82.40	79.13	80.32	84.55
SVB	87.25	81.27	83.58	90.93	80.83	79.47	79.61	83.43
Ours	**89.50**	**84.37**	**86.57**	**92.56**	**86.41**	**83.72**	**84.74**	**87.91**

Dataset	Wheat 2023				Wheat 2024			
DR	76.26	**81.89**	78.32	84.26	73.81	**84.79**	77.60	88.87
DT	83.68	68.34	72.13	85.66	82.73	71.23	75.33	91.81
GCN	79.23	76.82	77.91	86.19	86.52	74.78	79.16	92.96
SVB	82.26	78.79	80.32	87.85	85.35	76.69	80.20	93.03
Ours	**89.06**	79.67	**83.20**	**90.31**	**91.05**	78.07	**82.97**	**94.23**

**Notes.**

Ours refers to Phi+ResNet(finetune)+BiLSTM. Pre: average precision; Rec: average recall; F1: average F1-score; Acc: accuracy. The numbers in bold indicate the best performances of each dataset.

**Table 4:** The performances of five methods on “field” and “road” categories of the four trajectory datasets.

Dataset	Wheat 2021						Paddy					
	Field			Road			Field			Road		
	Pre	Rec	F1	Pre	Rec	F1	Pre	Rec	F1	Pre	Rec	F1
DR	86.47	96.19	91.03	69.21	34.81	45.59	79.62	79.33	79.17	54.43	56.61	54.41
DT	82.07	98.79	89.60	54.25	5.42	9.36	78.54	79.84	79.13	52.94	51.06	51.83
GCN	89.19	95.67	92.26	72.13	50.16	58.03	86.49	91.86	89.05	78.31	66.40	71.58
SVB	89.30	96.26	92.60	74.86	49.53	58.56	87.12	89.03	87.92	74.53	69.90	71.30
Ours	93.77	97.30	95.50	85.22	71.45	77.64	89.57	93.33	91.37	83.26	74.11	78.11

Dataset	Wheat 2023						Wheat 2024					
DR	93.78	85.91	89.67	58.74	77.88	66.97	97.19	90.06	93.49	50.43	79.52	61.72
DT	86.14	97.68	91.55	81.22	39.01	52.71	93.29	97.81	95.49	72.17	44.65	55.17
GCN	90.21	92.69	91.43	8.24	60.94	64.39	94.07	98.26	96.12	8.98	51.29	62.19
SVB	90.90	94.14	92.49	73.61	63.44	68.14	94.54	97.79	96.14	76.16	55.60	64.27
Ours	90.82	97.69	94.13	87.30	61.66	72.27	94.78	98.94	96.82	87.32	57.20	69.12

**Notes.**

Ours refers to Phi+ResNet(finetune)+BiLSTM. Pre, average precision; Rec, average recall; F1, average F1-score; Acc, accuracy.

Compared with our method which can simultaneously capture the motion characteristic and shape characteristic, some previous field-road classification methods did not use the shape characteristic, which hurts the classification performance. For example, DBSCAN+Rules utilized the shape characteristic by the rules, which is not an effective way. DT focused on only the motion characteristics. Meanwhile, some existing field-road classification methods attempted to utilize the shape characteristic in different ways. GCN used a graph convolutional neural network to encode the shape characteristic, which is an indirectly way. Although both SVB and our method detected the characteristic through an image, SVB did not utilize the rich general knowledge in the pre-trained model for its visual feature extraction and did not use detailed information on each point, leading to worse performance.

### Method analysis

In this section, we provide an in-depth analysis of our proposed method, which consists of three parts: (1) module analysis; (2) pretraining-finetuning paradigm analysis; (3) case study.

### Module analysis

As shown in Fig. 2, there are three components to carry out the feature extraction: physical encoder, visual encoder, and multi-view fusion encoder. To investigate the effect of these components, an ablation study was performed and the results are shown in Table 5 for the four datasets, respectively.

**Table 5:** The contribution analysis of different encoders on four trajectory datasets.

Dataset	Pre	Rec	F1	Acc	Pre	Rec	F1	Acc
	Wheat 2021				Paddy			
Phi+BiLSTM	81.06	70.11	72.82	86.34	76.78	73.82	74.74	79.80
ResNet(finetune)+BiLSTM	85.07	71.71	75.29	87.84	84.95	82.45	83.40	86.66
Phi+ResNet(finetune)+Cat	77.08	63.39	65.76	84.29	77.06	76.80	76.89	81.00
Phi+ResNet(finetune)+GRU	90.38	77.55	81.93	90.36	84.74	79.52	81.35	84.91
Phi+ResNet(finetune)+TE	88.32	82.35	84.94	92.51	84.55	82.94	83.67	86.64
Phi+ResNet(finetune)+BiLSTM	89.50	84.37	86.57	92.56	86.41	83.72	84.74	87.91

Dataset	Wheat 2023				Wheat 2024			
Phi+BiLSTM	70.63	74.62	72.10	79.97	71.86	70.76	71.29	88.79
ResNet(finetune)+BiLSTM	76.71	78.83	77.67	84.86	76.93	78.63	77.74	90.81
Phi+ResNet(finetune)+Cat	73.92	66.14	68.49	82.51	77.49	66.59	69.51	83.80
Phi+ResNet(finetune)+GRU	84.43	80.79	82.40	89.13	89.17	76.76	81.43	93.71
Phi+ResNet(finetune)+TE	88.13	79.46	82.77	89.99	83.26	78.18	80.43	92.76
Phi+ResNet(finetune)+BiLSTM	89.06	79.67	83.20	90.31	91.05	78.07	82.97	94.23

**Notes.**

**Phi+BiLSTM**: our feature extraction method removing the visual encoder; **ResNet(finetune)+BiLSTM**: our feature extraction method removing the physical encoder; **Phi+ResNet(finetune)+Cat**: our feature extraction method removing the BiLSTM network in the multi-view fusion encoder; **Phi+ResNet(finetune)+GRU**: our feature extraction method BiLSTM network in the multi-view fusion encoder replaced by gated recurrent unit (GRU) network; **Phi+ResNet(finetune)+TE (Transformer Encoder)**: our feature extraction method BiLSTM network in the multi-view fusion encoder replaced by Transformer-Encoder network; **Phi+ResNet(finetune)+BiLSTM**: our proposed feature extraction method.

First of all, we investigate the impact of the multi-view fusion feature extraction by removing either the physical encoder or the visual encoder from our proposed method, *i.e.,* ResNet(finetune)+BiLSTM and Phi+BiLSTM. In other words, only input features extracted by the physical encoder or by the visual encoder are fed to the multi-view fusion encoder. As we can see from Table 5, compared with our method, the overall performance of the two methods, ResNet(finetune)+BiLSTM and Phi+BiLSTM, dropped significantly. *E.g.*, for Phi+BiLSTM, there are 6.22%, 8.11%, 10.34%, and 5.44% accuracy score decreases on the four trajectory datasets, respectively. The performance drop indicates that both the physical encoder and the visual encoder are important.

Secondly, from Table 5, we observe that after removing the multi-view fusion encoder from our method, *i.e.,* Phi+ResNet(finetune)+Cat, the overall performance degrades significantly, *e.g.*, 8.27%, 6.91%, 7.8%, and 10.43% accuracy score decrease on the four trajectory datasets, respectively. Moreover, by replacing the BiLSTM network in the multi-view fusion encoder with a Transformer Encoder (TE) or gated recurrent unit (GRU) network (*i.e.,* Phi+ResNet(finetune)+TE or Phi+ResNet(finetune)+GRU), the method’s performance in four trajectory datasets exhibits different declines. This confirms that our BiLSTM-based multi-view fusion encoder can effectively fuse the two kinds of input features based on sequential relationships between points of a trajectory.

**Table 6:** The overall performances and time consumption of various visual encoders on four trajectory datasets.

Dataset	Pre	Rec	F1	Time	Pre	Rec	F1	Time
	Wheat 2021				Paddy			
Phi+ViT(finetune)+BiLSTM	90.40	83.65	86.45	625 s	86.76	84.15	85.18	57 s
Phi+MobileNet(finetune)+BiLSTM	85.37	81.43	83.21	501 s	84.41	83.17	83.64	38 s
Phi+ShuffleNet(finetune)+BiLSTM	88.30	76.82	80.94	520 s	83.15	80.91	81.62	39 s
Phi+ResNet(finetune)+BiLSTM	89.50	84.37	86.57	529 s	86.41	83.72	84.74	42 s

Dataset	Wheat 2023				Wheat 2024			
Phi+ViT(finetune)+BiLSTM	86.99	79.68	82.58	70s	83.41	82.80	83.06	59s
Phi+MobileNet(finetune)+BiLSTM	89.25	75.95	80.25	46s	82.39	80.25	80.92	33s
Phi+ShuffleNet(finetune)+BiLSTM	87.14	73.03	77.23	47s	83.20	74.98	76.76	35s
Phi+ResNet(finetune)+BiLSTM	89.06	79.67	83.20	50s	91.05	78.07	82.97	39s

**Notes.**

“Time” represents the average inference time of the model across ten experiments on the test set of each dataset. “ViT” represents the Vision Transformer network.

Finally, regarding the point-based image generation that produces an image for each trajectory point (Sec. Visual Encoder), the balance between visual model performance and inference speed is examined. To evaluate the impact of different visual encoders on performance and the time consumption of the inference process, the ResNet network used in our visual encoder is replaced with one of the following three networks: a popular Vision Transformer (ViT) network, and two lightweight networks (MobileNet and ShuffleNet). As shown in Table 6, compared to the ResNet network, both lightweight networks perform worse on all the datasets, and the Vision Transformer network achieves a slight performance improvement on the Paddy and Wheat 2024 datasets. However, the consumption of the Vision Transformer is approximately 1.5 times longer than that of the ResNet network, which will seriously affect the model’s inference speed. Therefore, this study opts for a ResNet-based visual encoder. Moreover, the different performances of the visual encoder between the Paddy and the other three Wheat datasets may be due to their different recording updated frequencies of GNSS signals (5 s *vs.* 30 s for the wheat and paddy harvesting trajectories, respectively). Compared with the wheat harvesting trajectory data, the paddy harvesting trajectory data has a much lower recording updated frequency, which results in less effective feature extraction of the physical encoder, and more reliance on the features provided by the visual encoder.

**Analysis of pretraining-finetuning paradigm:** To show the contribution of the pretraining-finetuning paradigm, the fine-tuned ResNet in our method was replaced by another model, ResNet or ResNet(pre-train), so as to generate two field-road classification models, Phi+ResNet+BiLSTM and Phi+ResNet(pre-train)+BiLSTM, respectively. Specifically, in Phi+ResNet+BiLSTM, a ResNet model is trained from a raw ResNet network with the images used in the fine-tuning process of our visual encoder, and then the trained ResNet model is used to extract visual features. In Phi+ResNet(pre-train)+BiLSTM, the pre-trained ResNet model is used to extract visual features. In other words, the neither the pre-training nor fine-tuning process is used in Phi+ResNet+BiLSTM, and no fine-tuning process is used in Phi+ResNet(pre-train)+BiLSTM. The results of the two methods are shown in Table 7 for the four trajectory datasets, respectively. We observe that after removing the model’s fine-tuning process from our visual encoder, *i.e.,* Phi+ResNet(pre-train)+BiLSTM, the overall performance degrades 6.71%, 1.54%, 1.39%, and 2.91% on F1-score for the four trajectory datasets, respectively. This reflects that the knowledge in our task-specific images has been successfully learned through the fine-tuning process (Fig. 4), and thus visual features specific to the field-road classification can be effectively extracted using the fine-turned ResNet model.

**Table 7:** The contribution analysis of the pretraining-finetuning paradigm on four trajectory datasets.

Dataset	Pre	Rec	F1	Acc	Pre	Rec	F1	Acc
	Wheat 2021				Paddy			
Phi+ResNet+BiLSTM	74.34	78.49	76.17	90.08	86.67	73.87	74.68	78.66
Phi+ResNet(pretrain)+BiLSTM	87.56	76.39	79.86	89.69	85.08	81.96	83.20	86.72
Phi+ResNet(finetune)+BiLSTM	89.50	84.37	86.57	92.56	86.41	83.72	84.74	87.91

Dataset	Wheat 2023				Wheat 2024			
Phi+ResNet+BiLSTM	79.11	76.27	77.40	88.24	78.20	72.96	75.20	90.98
Phi+ResNet(pretrain)+BiLSTM	86.08	79.00	81.81	89.27	81.75	78.60	80.06	92.41
Phi+ResNet(finetune)+BiLSTM	89.06	79.67	83.20	90.31	91.05	78.07	82.97	94.23

After removing the pretraining-finetuning paradigm from our visual encoder, *i.e.,* Phi+ResNet+BiLSTM, the overall performance drops 2.48%, 9.25%, 2.07%, and 3.25% on accuracy scores for the four trajectory data, respectively. This confirms that both the general knowledge learned by the pre-trained model and the task-specific knowledge learned through the fine-tuning process play an important role in our field-road classification method. Moreover, the paddy harvesting trajectory data can take more advantage of the pre-trained ResNet model, because it relies more on the features provided by the visual encoder.

**Figure 5:**
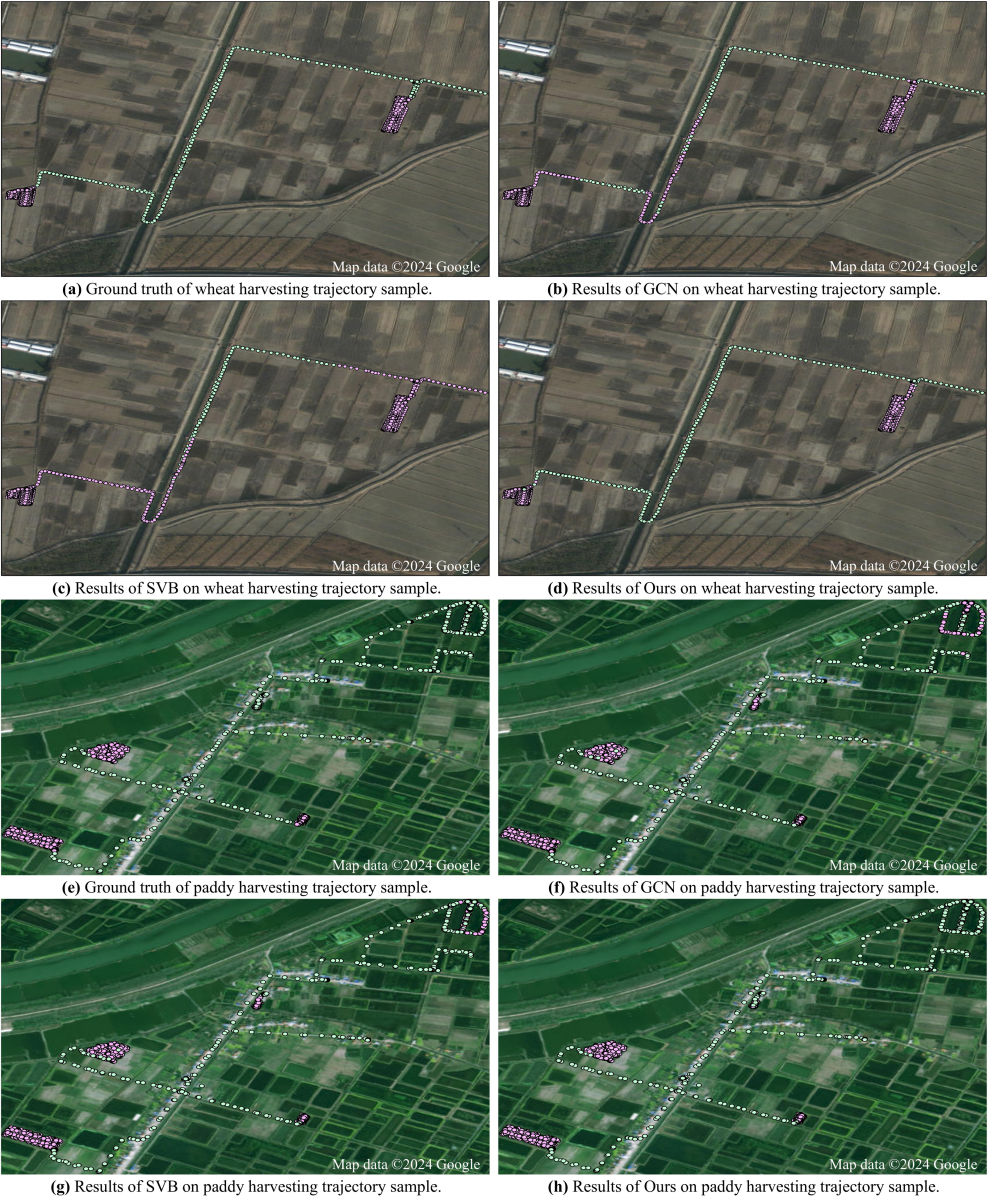
Results of different field-road classification methods for two harvesting trajectory samples. Satellite imagery©2024 Google Earth Engine; Map data©2024 Google.

**Case study:** To give an intuitive visualization, we show the results of two state-of-the-art field-road classification methods (*i.e.,* GCN and SVB) and our method on two trajectory samples: one is recorded during wheat harvesting and the other is recorded during paddy harvesting. From Fig. 5, we can see that on both samples, the GCN and SVB methods misidentify a lot of road trajectory points as field trajectory points. In contrast, our method can effectively reduce these mistakes. Through effectively extracting visual features from images generated for each trajectory point, our method can effectively distinguish characteristics between field and road trajectory points, leading to accurate classification.

## Conclusions

The research work presented in this paper solves the field-road classification task for agricultural vehicles. To capture the motion patterns specific to agricultural operations, this paper proposes a multi-view fusion feature extraction method which extracts two feature vectors from the physical and visual views to represent a trajectory point and then fuses these two representations based on sequential relationships between points of a trajectory. Particularly, to detect the shape characteristic in field, a colorful image is generated based on the information about the point and its neighboring points. Then, to effectively extract visual features, a fine-tuned ResNet model is developed using the pretraining-finetuning paradigm so that to utilize both the rich general knowledge learned by the pre-trained ResNet model and the task-specific knowledge learned through the fine-tuning process. According to the experiments on the four trajectory datasets, our proposed method, Phi+ResNet(finetune)+BiLSTM, achieves the best performance. Compared to the best baseline method (SVB), our method increases the F1-score by 2.99%, 5.13%, 2.88%, and 2.77% on the four trajectory datasets, respectively.

Moreover, there are many potential applications of the field-road classification. It can work as functionality in a machinery monitor system that provides a behavior analysis of agricultural vehicles, such as field efficiency analysis (Kortenbruck, Griepentrog & Paraforos, 2017), fuel consumption analysis (Lovarelli, Fiala & Larsson, 2018), and so on. It can provide statistics about the cost of the two activities (in-field and on-road) to optimize the scheduling of agricultural vehicles (Edwards et al., 2015). It would aid in relevant studies on cross-regional agricultural mechanization services (Zhang, Yang & Thomas, 2017).

The limitation of the proposed method is that it is a supervised learning method. Although massive amounts of agricultural vehicle trajectories have been collected, due to the time consumption in the process of annotating trajectory data, it is impossible to label large-scale trajectory data for supervising model learning. To address this problem, in the future, we will explore a self-supervised learning architecture to utilize large-scale unlabeled trajectory data for model training so as to further improve field-road classification models.

## Additional Information and Declarations

### Data Availability

The following information was supplied regarding data availability: The data is available at Zenodo: China Agricultural University. (2024). Field_road_dataset [Data set]. Zenodo. https://doi.org/10.5281/zenodo.10561232.
